# Letter to the Editor – Update from Ukraine: Development of the Cloud-based Platform for Patient-centered Telerehabilitation of Oncology Patients with Mathematical-related Modeling

**DOI:** 10.5195/ijt.2023.6562

**Published:** 2023-05-11

**Authors:** Kyrylo S. Malakhov

**Affiliations:** Microprocessor Technology Lab, V.M. Glushkov Institute of Cybernetics of the National Academy of Sciences of Ukraine

**Keywords:** Combat stress disorder, Digital health, Discrete and non-smooth optimization, Hybrid e-rehabilitation, Oncology, Subgradient algorithms, Telerehabilitation, Transdisciplinarity, Transdisciplinary research, Ukraine

## Abstract

This Letter to the Editor provides an update on the research from the Glushkov Institute of Cybernetics of the National Academy of Sciences of Ukraine. The Institute's research team in collaboration with Ternopil National Medical University began a new project called “Development of the cloud-based platform for patient-centered telerehabilitation of oncology patients with mathematical-related modeling.” The project is dedicated to the development of a hybrid cloud-based platform, and the creation on its basis of information technology for the telemedicine rehabilitation of cancer patients, and adapted for patients with combat stress disorder. The distinctive features of the proposed technology are a combination of artificial intelligence methods with accurate mathematical methods for optimization: developing mathematical models of problems of discrete, and non-smooth optimization, subgradient space transformation algorithms (to minimize non-smooth functions with tens of thousands of variables), and a method of global equilibrium search, etc.

April 22, 2023

Dear Ellen R. Cohn, PhD:

We are grateful that the May 2022 Special Issue of the *International Journal of Telerehabilitation* (O. V. Palagin et al., 2022) featured research from the Glushkov Institute of Cybernetics of the National Academy of Sciences of Ukraine. We appreciate the ongoing interest of the telerehabilitation professional community, and so offer this update.

At the beginning of 2022, the research team of our Institute again became one of the winners of the “Science for Safety and Sustainable Development of Ukraine” competition held by the National Research Foundation of Ukraine (Foundation). Our new project is titled “Development of the cloud-based platform for patient-centered telerehabilitation of oncology patients with mathematical-related modeling.” Due to Russia's military invasion, and aggression against Ukraine all funds were directed to the Ukraine army by the Foundation. Funding for all projects was suspended. In May 2023, the Foundation plans to resume project funding. The Institute's team in collaboration with Ternopil National Medical University began this project (Chaikovsky et al., 2023) and adapted its direction due to the martial law and the invasion of the Russian troops.

The project is dedicated to the development of the hybrid cloud-based platform along with information technology (IT) for the telemedicine rehabilitation (TMR) of cancer patients. This serves a wide range of specialists in physical therapy and rehabilitation medicine (PRM) in the sector “TMR of oncology patients.” The distinctive feature of the proposed technology is a combination of artificial intelligence (AI) methods with accurate mathematical methods for optimization: developing mathematical models of problems of discrete and non-smooth optimization, subgradient space transformation algorithms (to minimize non-smooth functions with tens of thousands of variables), and methods of global equilibrium search, etc. The development of sequential and parallel algorithms for solving complex optimization problems practiced at the modern supercomputer complex of the Institute is a specialization of a group of project authors. All planned tasks focused on: the prediction of the current, and chronic patient's condition, strategies for health intervention in the rehabilitation process, clustering of oncology patients, funds distribution for treatment (at the state, and other levels), etc. The technological platform of interactive knowledge will be implemented, as a component set of services. Each service is supported by an ontology-related scheme (O. Palagin et al., 2014), and provides the dynamic formation of the ordered functionality by means of ontology-driven service-oriented architecture (O. V. Palagin et al., 2018). Cognitive services of the basic information technology provide the implementation of information and analytical platforms capable of efficiently integrating and processing all of the information that is formed in spatially distributed network sources that it continuously receives and processes by all rehabilitation specialists.

**Strategic areas of the project's research activity are as follows**. The project is focused on improving, and applying new promising medical technologies that meet the criteria of global novelty: *modeling of pathological processes, and development of principles of experimental therapy, and prevention.*

The main tasks of the project include:

- development of the hybrid cloud-based platform for telemedicine rehabilitation with all its components (servers, databases, etc.);
- creation of a set of TMR tools (biomonitoring, and diagnostic devices, tools for TMR procedures support for rehabilitation);
- development of the application of mathematical models and methods of prediction and optimization in relation to the processes of TMR and the organization of the IT architecture component;
- approbation of the platform in the TMR center;
- creation and approbation of the training course “Methods and services of TMR for oncology patients in the case of breast cancer patients” for PRM experts;
- development of the TMR's direction in Ukraine through the creation of a branch of the association of intelligent and IT's problems in TMR, bringing together scientists, and developers of new equipment and technologies in the field of TMR.

**Novel aspects of the project are as follows**. The general strategy underlying the project is a comprehensive approach based on the development of accurate mathematical-based methods and technological services that underlie the problem of effective telerehabilitation of cancer patients and addresses all its main components:

- science-based regulatory initiatives, and legal aspects;
- methodological basis of telerehabilitation; the new online ITs to support all telerehabilitation processes (O. V. Palagin et al., 2022), based on the wide purposeful use of AI (O. V. Palagin et al., 2020);
- effective mathematical methods of big data analysis, modeling and creation of scenarios for the prediction and optimization of the whole set of rehabilitation procedures and their routes;
- usage of already team tested system tools and technologies via experience in the development of rehabilitation systems.

Thus, a complex productive triad is being formed, some components of which the team already has extensive experience: rehabilitation methodology, cognitive intelligent ITs, mathematical-related methods of modeling and optimization of the rehabilitation processes throughout its life cycle.

The following components are key to ensuring the high efficiency of the telerehabilitation process and the creation in Ukraine of a high-quality system of evidence-based medicine in the field of telerehabilitation of cancer patients to improve their quality of life:

- creation of a united general model, and its exact mathematical substantiation;
- decision of a complex of the optimization problems on all space of the set of rehabilitation tasks;
- relying on modern achievements in the fields of physiotherapy, psychotherapy, cognitive-behavioral therapy and art therapy in combination with biofeedback, and the patient-centered paradigm.

To solve a set of optimization problems, new modifications of the following methods developed at the Glushkov Institute of Cybernetics will use:

- a method of global equilibrium search;
- approximate stochastic method of obtaining qualitative solutions of boolean optimization problems of large dimension;
- subgradient methods with space transformation, which are in effective competition with modern effective methods of minimizing non-smooth functions.

To solve linear, integer, and nonlinear programming problems, modern software (Gurobi, CPLEX, IPOPT) will be used.

**Expected results of the project** are as follows:

- Scientific and technical products created as a result of the project: a hybrid cloud-based telerehabilitation platform (CTP) which consists of a set of functional subsystems (FS) and services;
- FS for supporting telerehabilitation activities based on the International Classification of Functioning, Disability and Health ICF; diagnostic FS; reference FS; predictive and analytical FS for evaluating the effectiveness of the telerehabilitation process; general medical FS; FS for displaying the telerehabilitation process;
- FS for network information exchange and a library of video sessions of rehabilitation procedures.

The project's research team was selected to cover all three components of the productive triad. The **core group** represents the world-famous School of Academician Viktor Glushkov in the field of systems analysis and optimization, IT, and AI systems: Petro Stetsyuk (project supervisor, DSc, specialist in linear and nonlinear programming, mathematical and software support for applied optimization problems); Ivan Sergienko (Academician of the National Academy of Sciences of Ukraine, DSc, Professor, Cybernetic Center General director, specialist in computer science, computational mathematics, optimization theory, system analysis and mathematical modeling, software for electronic computers; author of more than 800 scientific works, including 55 monographs, more than 20 patents); Oleksandr Palagin (Academician of the National Academy of Sciences of Ukraine, DSc, Professor, Deputy Director, Head of Microprocessor Technology Lab); Mykola Budnyk (DSc, specialist in the processing of biomedical signals, and the development of biomedical equipment); Denys Symonov (Junior research fellow, an expert in dynamic mathematical modeling systems); Kyrylo Malakhov (MSc, Researcher, Backend developer, DevOps engineer, member of the expert Subgroup on Technical Issues and Architecture of Telemedicine within the Interdepartmental Working Group for the Development of the Concept of Implementation of Telemedicine in Ukraine).

The **clinical group** consists of certified physicians in physical and medical rehabilitation, an oncologist, and organizers in the field of rehabilitation: Oleksandr Vladymyrov (professor, Honored Doctor of Ukraine, head of the department of physical and rehabilitation medicine Shupyk National Medical Academy of Postgraduate Education); Tetiana Semykopna (PhD, MD, Physical Medicine and Rehabilitation (PM&R); Oksana Syvak (professional manager of projects and clinical centers in different countries: EU, USA, Canada, Australia, etc. She has therapeutic experience in clinical oncology (breast cancer, lymphoma, NSCLC), pulmonology, gynecology, neurology, endocrinology, etc.; and since 2021 is Deputy Director of National Cancer Institute of Ministry of Health of Ukraine). Dmytro Vakulenko (Professor, DSc, PhD, Head of the Department of Medical Informatics, Horbachevsky Ternopil National Medical University, He specializes in developing the methods and tools for processing of biomedical data, including simulation algorithms for analysis; developing the elements of medical information and telecommunication systems).

This study would not have been possible without the financial support of the National Research Foundation of Ukraine. Our work was funded by Grant contract with application ID: 2021.01/0136.

Sincerely, Kyrylo Malakhov (On behalf of the Institute's research team)
